# A Novel Insight into Functional Divergence of the MST Gene Family in Rice Based on Comprehensive Expression Patterns

**DOI:** 10.3390/genes10030239

**Published:** 2019-03-20

**Authors:** Xiaolong Deng, Baoguang An, Hua Zhong, Jing Yang, Weilong Kong, Yangsheng Li

**Affiliations:** State Key Laboratory for Hybrid Rice, College of Life Sciences, Wuhan University, Wuhan 430072, China; 2009202040085@whu.edu.cn (X.D.); abg803517@163.com (B.A.); zhonghua0103@whu.edu.cn (H.Z.); piaoxcy@hotmail.com (J.Y.); Weilong.Kong@whu.edu.cn (W.K.)

**Keywords:** MST family, rice, *Oryza sativa*, functional divergence, expression analysis, qRT-PCR

## Abstract

Sugars are critical for plant growth and development as suppliers of carbon and energy, as signal molecules, or as solute molecules for osmotic homeostasis. Monosaccharide transporter (MST) genes are involved in various processes of plant growth and development as well as in response to abiotic stresses. However, the evolution and their roles of MST genes in growth and development and in coping with abiotic stresses in rice are poorly known. Here, we identified 64 MST genes in rice genome, which are classified into seven subfamilies: STP, PLT, AZT, ERD, pGlcT, INT, and XTPH. MST genes are not evenly distributed between chromosomes (Chrs) with a bias to Chr 3, 4, 7, and 11, which could be a result of duplication of fragments harboring MST genes. In total, 12 duplication events were found in the rice MST family, among which, two pairs were derived from fragmental duplications and ten pairs were from tandem duplications. The synonymous and nonsynonymous substitution rates of duplicate gene pairs demonstrated that the MST family was under a strong negative selection during the evolution process. Furthermore, a comprehensive expression analysis conducted in 11 different tissues, three abiotic stresses, five hormone treatments, and three sugar treatments revealed different expression patterns of MST genes and indicated diversified functions of them. Our results suggest that MST genes play important roles not only in various abiotic stresses but also in hormone and sugar responses. The present results will provide a vital insight into the functional divergence of the MST family in the future study.

## 1. Introduction

Sugars are critical for plant growth and development as suppliers of carbon and energy, signal molecules, or as solute molecules for osmotic homeostasis [1,2,3,4]. The adequate production, storage, and transport of sugars are tightly regulated both spatially and temporally [2,5]. In plants, various sugar transporters, including the SWEET (sugar will eventually be exported transporters) family and major facilitator superfamily (MFS) [6,7,8], are engaged in the transport of sugars from source (such as mature leaves) to sink organs (e.g., roots, seeds, and other reproductive organs) [6,7,8]. MFS can be further divided into the sucrose transporter (SUT) family and monosaccharide transporter (MST) family according to their specified substrates. MSTs are integral membrane proteins, which are involved in the transmembrane transport of a wide range of monosaccharides [1,4,6].

Genome-wide identification of MST family has been conducted in many plants including *Arabidopsis* [9,10,11], *Vitis vinifera* [12], *Nicotiana tabacum* [13], *Solanum lycopersicum* [14,15], *Medicago truncatula* [16], and *Rosa hybrid* [17]. Generally, MST family can be classified into seven subfamilies, STP, PLT, XTPH, INT, AZT, pGlcT, and ERD, based on their sequence features and substrate specificities [18,19]. In *Arabidopsis*, the identified STP proteins (STPs) are H^+^/hexose cotransporters locating on plasma membranes. Most STPs exhibit a broad spectrum of absorption characteristics of substrates [6,10]. For example, AtSIP1, AtSIP2, AtSIP3, AtSIP4, AtSIP6, and AtSIP11 are able to transport glucose, xylose, mannose, and galactose in different affinities, while they cannot transport fructose [2]. In contrast, AtSTP6, AtSTP13, and OsMST4 could transport fructose but do not transport pentose, xylose, and ribose [10,12,20]. However, a few STP proteins show substrate specificity in transport. For instance, AtSTP9 specifically transports glucose and AtSTP14 exhibits specific transport of galactose [21,22]. Moreover, PLT proteins (PLTs) transport not only polyols but also monosaccharides [23,24]. In XTPH proteins (XTPHs, also called VGTs), AtVGT1 can absorb glucose but not xylose. AtVGT1 and AtVGT2 are both H^+^/glucose reverse transporters localized on the tonoplast, which are involved in the transport and storage of monosaccharides in vacuoles [9,11]. AZT proteins (ATZs, also called TMT) were localized on tonoplast, too. Previous studies reported that the sucrose uptake capacity of vacuoles was reduced in the attmt1/attmt2 mutant of *Arabidopsis*, suggesting that TMTs are involved in transporting sucrose on the tonoplast [25]. pGlcT proteins (pGlcTs) was reported to transport glucose [26]. In *Arabidopsis*, ERD (ERD6s, also called SFPs) is the biggest subfamily in MST family, and the first identified ERD protein (AtERD6) was induced by drought and low temperature [27]. Among them, AtSFP1 and AtSFP2 are stress-inducible facilitated diffusion transporters and differ greatly in spatiotemporal expression [28]. Taken together, MST family genes play important roles in plant growth and development, as well as in responses to biotic and abiotic stresses.

Several MST genes had been studied in rice. For example, Toyofuku et al. (2000) cloned and analyzed three MST genes in rice, *OsMST1*, *OsMST2*, and *OsMST3*, and found that OsMST3 protein is able to transport some monosaccharides via an energy-dependent H^+^ co-transport manner [29]. Furthermore, *OsMST5* is involved in pollen development in rice and its monosaccharide transport activity was characterized by heterologous expression analysis [30]. In addition, *OsMST4* was ubiquitously expressed and had the capability of transporting fructose, galactose, mannose, and glucose [22]. In 2008, Wang et al. demonstrated that *OsMST6* was a broad-spectrum monosaccharide transporter and its expression was induced by salt stress and sugars [31]. *OsTMTs* was involved in vacuolar sugar transport [2]. Moreover, *OsGMST1* was up-regulated under salt stress conditions and knock down of this gene conferred hypersensitivity to salt stress in rice [32]. The evolution and functional divergence analysis of OsMSTs have been systematically elucidated in 2007 [19].

While several of these genes have been well studied in various organisms, the functions of the most MST family remain unclear to date. In this study, to interpret the possible physiological role of this family, we conducted a comprehensive analysis of expressions of MST genes in different tissues and under different treatments. Additionally, a systematic bioinformatics analysis was performed, including phylogenetic reconstruction, chromosome location, structural analysis of genes and proteins, gene duplication events, and the synonymous (Ks) and nonsynonymous (Ka) substitution rates of duplicate gene pairs (Ka/Ks ratios). This study provides vital insights into the evolution of the MST family in rice and leads to a better understanding of the MSTs functions.

## 2. Materials and Methods

### 2.1. Plant Materials and Treatments

Rice ‘9311’ (*O. sativa* ssp. *indica*) was used for quantitative Real-Time PCR (qRT-PCR). After two days of germination in water at 37 °C, seeds were transferred into vermiculite saturated with ddH_2_O for further growth. All seedlings were grown at 26 °C, with a daily photoperiodic cycle of 16 h light and 8 h dark, with 60% relative humidity.

In this study, 11 tissue samples were collected for tissues specific expression analysis as the previous study [33]. They were calli (Cal, induced 30 days before subculture), grouting seed (GS, embryo and endosperm at 12–15 days after flowering), root (Rt, 12 day old seedlings), shoot (Sh, 12 day old seedlings), flag leaf (FL, 1 week after heading), sink leaf (SL, unexpanded flag leaves harvested approximately 3 weeks before heading), sink flag leaf sheath (SinkFLS, flag leaf sheaths harvested from plants 1 week before heading), source flag leaf sheath (SourceFLS, flag leaf sheaths harvested from plants 1 week after heading), node (Nd, the first node on the top at panicle stage), internode (InterN, part between the first node and the second node on the top at panicle stage), and panicle (Pan5, panicle grown to the length of 5 cm). Three biological replicates were performed, with samples of each were collected from 15 plants. All the samples were triturated immediately with liquid nitrogen and stored at −80 ℃ before they were used for RNA extraction.

12 days old seedlings were treated with indole-3-acetic acid (IAA, 50 μM), 6-benzylamino purine (6BA, 25 μM), abscisic acid (ABA, 100 μM), gibberellic acid (GA, 100 μM), and salicylic acid (SA, 100 μM) by spraying. Samples (leaves) were collected at 0, 1, 3, 6, and 12 h. As to salt, osmotic, and drought treatments, the roots of 12 days old seedlings were rinsed, followed by the immediate immersion in NaCl solution (200 mM), PEG 6000 solution (20%, *w*/*v*), and air. Samples (leaves) were also collected at 0, 1, 3, 6, and 12 h. Three biological replicates were produced for every treatment, each of which was collected from 12 seedlings and pooled together. These samples were triturated immediately with liquid nitrogen, and stored at −80 °C for further use. The sugar treatments were also performed similarly with the NaCl, PEG 6000 solutions in salt stress treatments were replaced by 2% sucrose, glucose, and fructose solutions.

### 2.2. Identification of the MST Genes

MST genes were identified using a Hidden Markov model (HMM) and BLAST homology searches [34]. The reference genome sequence and annotations were downloaded from MSU 7.0 (http://rice.plantbiology.msu.edu). *Arabidopsis* MST protein sequences were downloaded from the TAIR database (https://www.arabidopsis.org/). The *Arabidopsis* MST proteins were queried against rice genome using BLASTP (e-value, e-5) to search for rice MST genes [8,35]. PF00083 was downloaded from Pfam (http://pfam.xfam.org/) and used to search the rice proteins database using HMMER 3.0 software (http://hmmer.org/). Finally, SMART (http://smart.embl-heidelberg.de/) and Pfam (http://pfam.xfam.org/search/sequence) were utilized to verify these sequences [34,35].

### 2.3. Phylogenetic Analysis, Gene Structure, and Conserved Motifs

Multiple sequences alignments were performed using ClustalW with default parameters (protein weight matrix was Gonnet). MEGA 6.0 (The Pennsylvania State University, PA, USA, https://www.megasoftware.net/) was used to construct a Neighbor-Joining (NJ) phylogenetic tree of rice MST genes with 1000 bootstrap replicates [8,35,36]. Conserved motifs of MST proteins were identified by Multiple Expectation Maximization for motif Elicitation (MEME, http://meme-suite.org/tools/meme) tool with the maximum number setting to 12 [8,35]. Gene structures (exon/intron) of all MST genes were analyzed using GSDS 2.0 (http://gsds.cbi.pku.edu.cn/). Finally, TBtools was used to visualize the phylogenetic tree, gene structure, and conserved motifs of MST genes [37].

### 2.4. Chromosomal Locations, Gene Duplication Events, and Ka/Ks Values

Coordinates on the reference genome sequence of all MST genes were obtained from the genome annotation file. Gene duplication events were identified using the ‘duplicate_gene_classifier’ script with the default parameters, in MCScanX software (University of Georgia, GA, USA, http://chibba.pgml.uga.edu/mcscan2/) [38]. Locations of all MST genes were mapped by TBtools [34] and gene duplication events were visualized using Circos software (http://circos.ca/) [38,39]. In addition, the synonymous (Ks) and nonsynonymous (Ka) substitution rates of MST duplicate gene pairs were estimated using DnaSP 5.0 (http://www.ub.edu/dnasp/) [40]. The divergence time of each duplication event in MST family was calculated by T = Ks/(2 × 9.1 × 10^−9^) × 10^−6^ million years ago (Mya) [38].

### 2.5. Expression Analysis of MST Genes by qRT-PCR

All RNA extraction and reverse transcription were performed by TRIzol Ragent (Invitrogen, Beijing, China) and PrimeScript RT reagent Kit (TakaRa, Dalian, China) according to instruction manuals. qRT-PCR analyses were performed in a 96-well plate on Bio-Rad CFX96 real time PCR system (Bio-Rad, Hercules, CA, USA). The qRT-PCR reaction (10 μL) was formulated using SYBR Green Master Mix reagent (Bio-Rad, Hercules, CA, USA). All the thermal cycles were as follows: 95 °C for 5 min; 40 cycles of 95 °C for 10 s, 58–62 °C for 10 s, 72 °C for 15 s; then melt curve from 65–95 °C. Three biological replicates (from three independent RNA extractions) of each treatment were used for qRT-PCR and three technical replicates from the same RNA extraction were used for each biological replicate. The amplification specificity of primers was verified by melting curve analysis. *Actin* gene was used as an internal control (Primer F: TGCTATGTACGTCGCCATCCAG; Primer R: AATGAGTAACCACGCTCCGTCA). The relative expression levels of MST genes were calculated by the 2^−ΔΔCT^ method (Table S1) [41].

### 2.6. Expression Correlation between MST Paralogs under Hormone and Abiotic Stress Treatments

Expression profiles of MST duplicate gene pairs were manually gathered from qRT-PCR data. Using CORREL (array1, array2) function in Excel, we calculated the correlation coefficient between each pair. According to earlier studies, significant values can assess the degree of expression diversity [42,43]. Generally, r < 0.3, 0.3 < r < 0.5, and r > 0.5 mean divergence, ongoing divergent, and non-divergence, respectively [42,43].

## 3. Results

### 3.1. Identification, Classification, Chromosome Locations, Duplication Events, and Selection Pressure of the MST Genes

**Figure 1:**
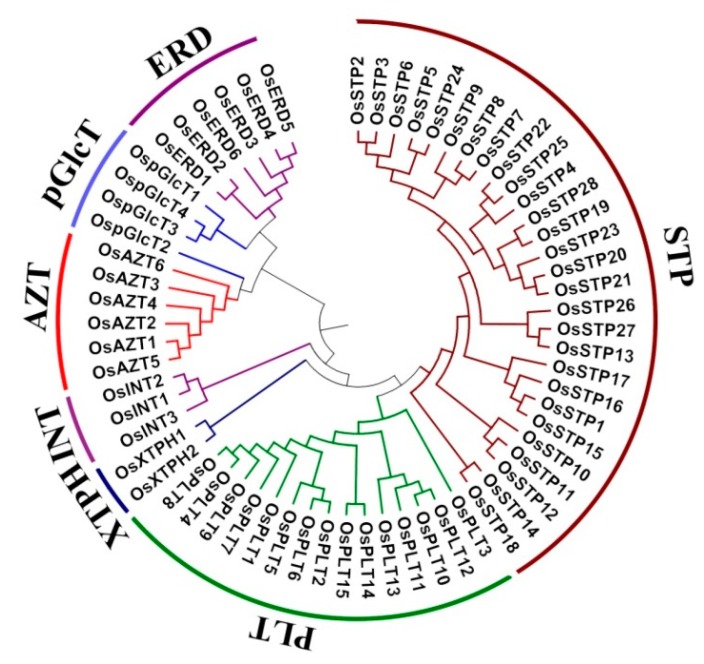
Phylogenetic tree of the monosaccharide transporter (MST) protein sequences in rice. Multiple sequence alignment is conducted by ClustalW. Phylogenetic tree is established by Neighbor-Joining (NJ) clustering method, with 1000 Bootstrap replicates, using MEGA 6.0 software (The Pennsylvania State University, PA, USA). Different colors of arcs indicate different subfamilies.

**Figure 2:**
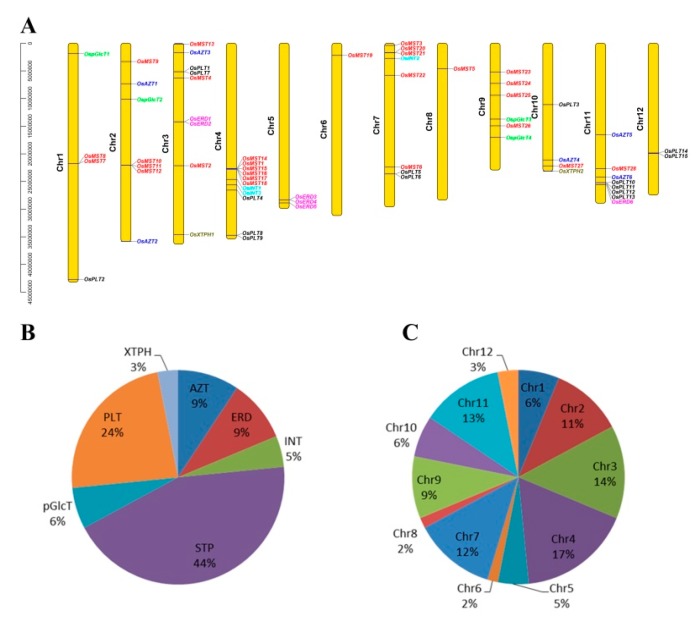
Chromosomal locations of the monosaccharide transporter (MST) genes. (**A**) Different colors of genes represent the members from different subfamilies. (**B**) Pie charts of different sizes indicate the ratio of each subfamily. (**C**) Pie charts of different sizes indicate the ratio of each chromosome.

A total of 64 MST genes were identified and grouped into seven subfamilies (Figure 1 and Table 1): 28 STPs (44%), 15 PLTs (24%), six AZTs (9%), six ERDs (9%), four pGlcTs (6%), three INTs (5%), and two XTPHs (3%) (Figure 2B, Figure S1). These genes are widely dispersed across different chromosomes with a bias to Chr 4, 3, 7 and 11. Among them, 11 (17%), nine (14%), eight (12%), and eight (12%) genes were located on Chr 4, 3, 7 and 11, respectively. Furthermore, Chr2, Chr9, Chr1, Chr10, Chr5, and Chr12 had seven, six, four, four, three, and two genes, respectively, while only one gene was found in Chr6 and Chr8 (Figure 2A,C). In short, all MST genes were unevenly distributed across all 12 chromosomes. Similar uneven distribution patterns of the MST genes were also found in *Arabidopsis* [9,10,11], *Vitis vinifera* [12], and *Nicotiana tabacum* [13].

**Figure 3:**
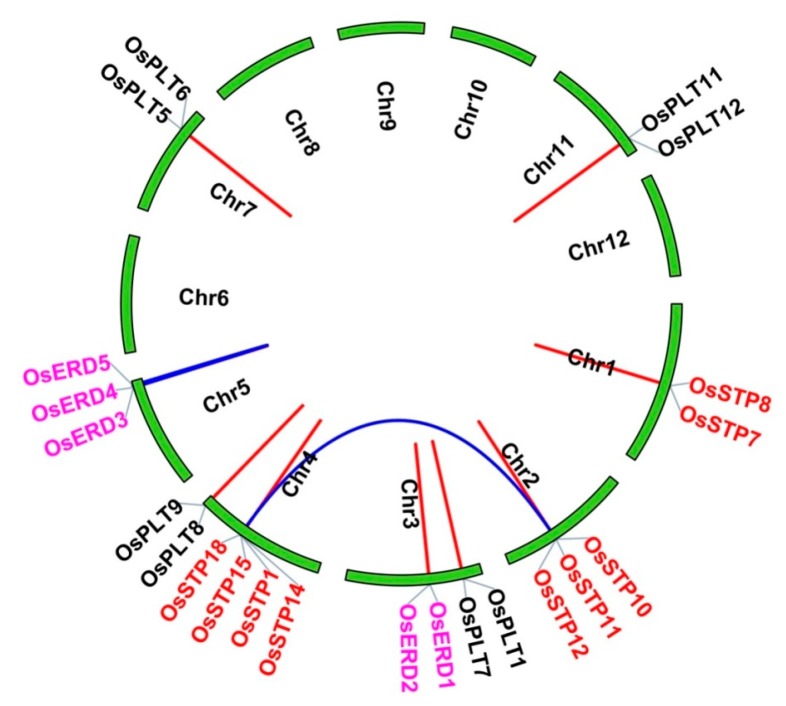
Duplication events of the monosaccharide transporter (MST) genes in rice. Different colors of genes represent the members from different subfamilies. The duplicate gene pairs of WGD or segmental/tandem events are shown with blue/red lines.

In addition, we observed that many genes from the same subfamily formed tandem gene clusters, such as STP cluster in Chr1, Chr2, Chr4, and Chr7, as well as ERD cluster in Chr3 and Chr5 (Figure 2A). We conjectured that gene clusters may be generated from tandem duplications and segmental duplications. Thus, MST duplicate gene pairs were evaluated using MCScanX. As a result, two segmental duplication events and ten tandem duplication events were found (Figure 3 and Table 2). Interestingly, we noticed that duplication events were only found in STP, ERD, and PLT subfamilies (Figure 3 and Table 2).

Next, Ka/Ks values of MST duplicate gene pairs were calculated to evaluate the driving force underlying the MST gene’s evolution. Ka/Ks > 1 means a positive selection, Ka/Ks < 1 means a negative selection, and Ka/Ks = 1 means a neutral selection [8,38]. The results showed that Ka/Ks values of MST duplicate gene ranged from 0.1603–0.7193, indicating that MST family was under a strong negative selection during the evolution process (Figure 3). The divergence times of duplication events were estimated from 1.80–18.08 Mya (Figure 3).

### 3.2. Gene Structure and Conserved Motifs

**Figure 4:**
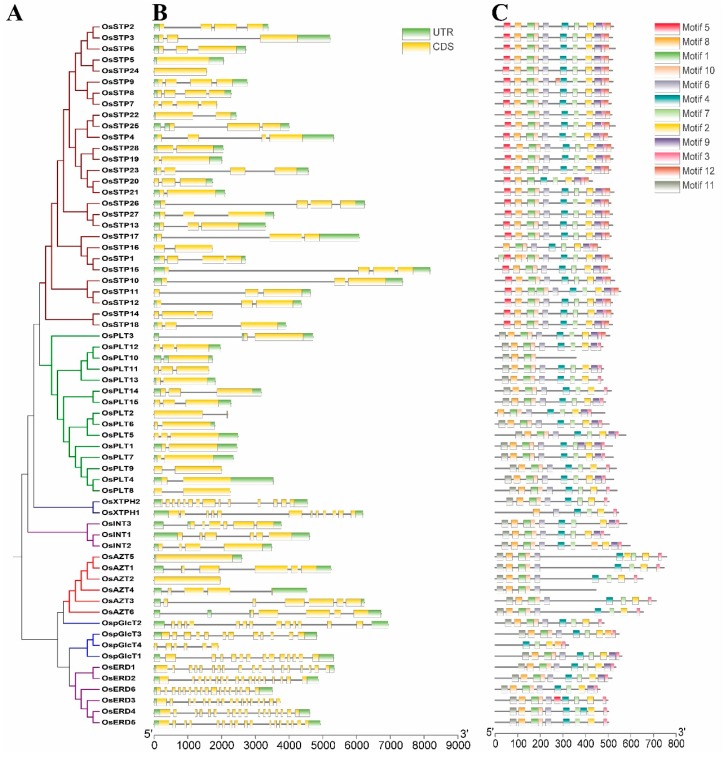
Phylogenetic tree (**A**), exon/intron structure (**B**), and motif compositions (**C**) of the monosaccharide transporter (MST) genes in rice. A: The Neighbor-Joining (NJ)-Phylogenetic trees are made in the same method with Figure 1B,C: The widths of grey bars at the bottom display relative lengths of genes and proteins. Green boxes and grey lines in B represent exons and introns, respectively. Different boxes in C represent different motifs.

We also analyzed the gene structures and conserved motifs in rice MST genes (Figure 4), as earlier studies suggested the diversity of gene structure and protein sequences as an important driving force of multigene families’ evolution [29,34,38]. Analysis of gene structure showed that members of the XTPH, ERD, and pGlcT subfamilies had more exons than other subfamilies in coding sequence (CDS) regions, while PLT subfamily had fewer exons. On the other hand, large variations of exon numbers were observed in STP, INT, and AZT subfamilies (Figure 4B). As expected, the motifs arrangement was strongly conserved in the same subfamilies, while there were big differences between different subfamilies (Figure 4C).

### 3.3. Expression Analysis of MST Genes in Various Tissues

**Figure 5:**
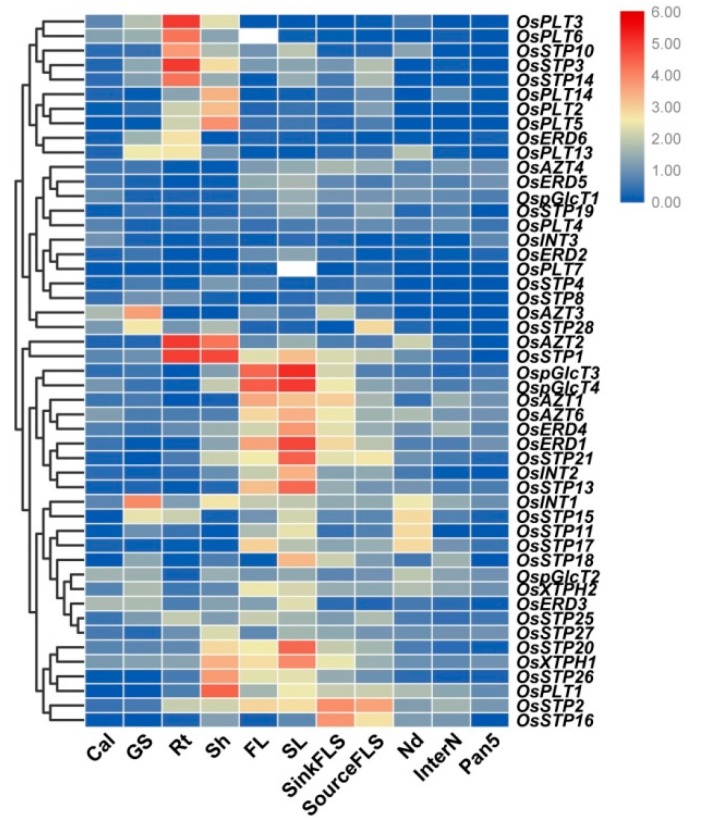
Expression profiles of the MST genes in 11 different tissues by qRT-PCR. Cal: calli from 30 days before subculture; GS: grouting seed at 12–15 days after flowering; Rt: root of 12 days old seedlings; Sh: shoot of 12 days old seedlings; FL: flag leaf at 1 week after heading; SL: unexpanded flag leaves harvested approximately 3 weeks before heading; SinkFLS: flag leaf sheaths harvested from plants 1 week before heading; SourceFLS: flag leaf sheaths harvested from plants 1 week after heading; Nd: the first node on the top at panicle stage; InterN: internode, part between the first node and the second node on the top at panicle stage; Pan5: the length of 5 cm of panicle. The color scale represents relative expression levels based on the 2^−ΔΔCT^ method: red indicates a high level and green represents a low level of transcript abundance.

To further characterize the potential function of MST genes, a comprehensive expression analysis of MST genes in 11 tissues was analyzed using qRT-PCR. In the present study, we found diversified expression pattern between different MST genes in rice. For example, several genes were lowly expressed in all tested tissues, namely *OsAZT4*, *OsERD5*, *OspGlcT1*, *OsSTP19*, *OsPLT4*, *OsINT3*, *OsERD2*, *OsPLT7*, *OsSTP4*, and *OsSTP8*, while the remaining MST genes showed relatively higher expressions in some specific tissues (Figure 5). For example, *OsPLT3*, *OsPLT6*, *OsSTP10*, *OsSTP3*, and *OsSTP14* had the predominant expression in roots; *OsPLT14*, *OsPLT2*, and *OsPLT5* showed higher expressions in shoots; *OsAZT2* and *OsSTP1* were highly expressed in both roots and shoots. *OspGlcT3*, *OspGlcT4*, *OsAZT1*, *OsAZT6*, *OsERD4*, *OsERD1*, *OsSTP21*, *OsINT2*, and *OsSTP13* showed relatively high expression in flag leaf at 1 week after heading, sink leaf at heading stage, and sink flag leaf sheath at heading stage. *OsINT1*, *OsATZ3*, and *OsSTP28* were highly expressed in grouting seed at 12–15 days after flowering. Besides, *OsSTP20*, *OsXTPH1*, *OsSTP26*, and *OsPLT1* showed relatively high expression in shoots of 12-days-old seeding, in flag leaf at 1 week after heading and sink leaf at heading stage. *OsSTP2* and *OsSTP16* had advantage expressions in sink flag leaf sheath at the heading stage and source flag leaf sheath at 1 week after heading. In addition, we compared the expression of duplicate gene pairs. Different expression patterns were found in all the gene pairs (Figure 5). We particularly noticed that *OsSTP14* and *OsSTP1*, which were tandem duplicated genes, showed similar expression patterns in various tissues, except *OsSTP1* was highly expressed in flag leaf and leaf (Figure 5).

### 3.4. Expression Analysis of MST Genes under Abiotic Stresses

**Figure 6:**
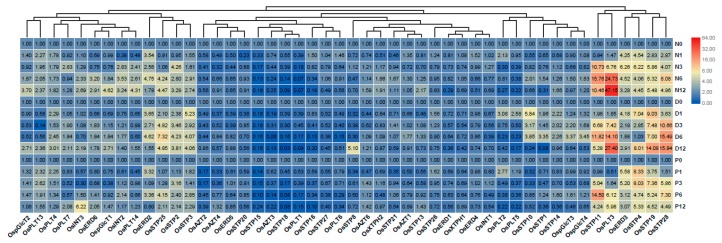
Expression changes of the MST genes under three abiotic stresses by qRT-PCR. N0, N1, N3, N6, and N12 represent 0 h, 1 h, 3 h, 6 h, and 12 h after 200 mM NaCl solution treatment. D0, D1, D3, D6, and D12 represent 0 h, 1 h, 3 h, 6 h, and 12 h after roots exposed to the air. P0, P1, P3, P6, and P12 represent 0 h, 1 h, 3 h, 6 h, and 12 h after 20% PEG6000 solution treatment. The fold values were calculated by the 2^−ΔΔCT^ method. The heat map was made based on the fold changes. The color scale represents fold changes: red indicates high up-regulation and green represents low up-regulation.

We further performed qRT-PCR analyses to detect the transcript change levels of MST genes under different abiotic stresses, including salt stress, osmotic stress, and drought stress. In this study, the fold changes >1.5 was considered to be a significant difference for the gene under treatments. We found that most MST genes were downregulated, while some others were upregulated under the tested abiotic stresses (Figure 6). *OsERD2*, *OsSTP25*, *OsSTP2*, *OsSTP3*, *OsSTP11*, *OsPLT3*, *OsERD3*, *OsSTP4*, *OsSTP19*, and *OsSTP28* were significantly up-regulated under three abiotic stresses (Figure 6). However, some genes exhibited stress-specific expression profile. For instance, *OsERD6*, *OspGlcT2*, *OsINT2*, and *OsPLT14* showed higher up-regulation under salt stress than under osmotic and drought stresses. *OsSTP10*, *OsSTP1*, *OsSTP14*, OspGlcT3, and *OspGlcT4* were higher up-regulated under osmotic stress than under salt and drought stresses. *OsPLT4* showed higher up-regulation under salt and drought stress than under osmotic stresses. *OsPLT13* were higher up-regulated under salt and osmotic stress than under drought stresses (Figure 6).

### 3.5. Expression Analysis of MST Genes under Hormone Treatments

**Figure 7:**
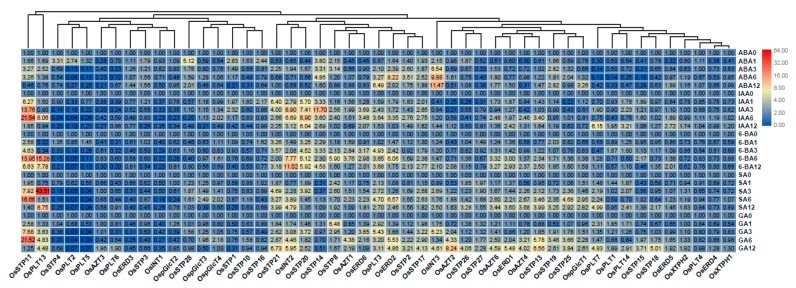
Expression changes of the MST genes under ABA, IAA, 6-BA, SA, and GA treatments by qRT-PCR. 0, 1, 3, 6, 12 represents 0 h, 1 h, 3 h, 6 h, and 12 h after hormone treatment. The fold values were calculated by the 2^−ΔΔCT^ method. The heat map was made based on the fold changes. The color scale represents fold changes with red indicating high and green indicating low up-regulation.

To our knowledge, the function of the MST family under hormone treatments was not well-documented in rice. In this present study, we found that expression patterns of MST genes differed under five different hormone treatments (Figure 7). We found that *OsSTP11*, *OsERD2*, and *OsPLT3* were up-regulated under ABA, IAA, 6-BA, SA, and GA treatments. However, *OsINT3* showed higher up-regulation under ABA treatment than under other treatments (Figure 7). *OsSTP21* and *OsINT2* were higher up-regulated under IAA, 6-BA, SA, and GA treatments than under ABA treatment (Figure 7). *OsSTP20* showed higher up-regulation under IAA, 6-BA, and GA treatments than under ABA and SA treatments (Figure 7). *OsSTP14* showed higher up-regulation under ABA, IAA, and 6-BA treatments than under SA and GA treatments. *OsPLT7* were higher up-regulated under SA and GA treatments than under ABA, IAA, and 6-BA treatments (Figure 7).

### 3.6. Expression Analysis of MST Genes under Sugar Treatments

**Figure 8:**
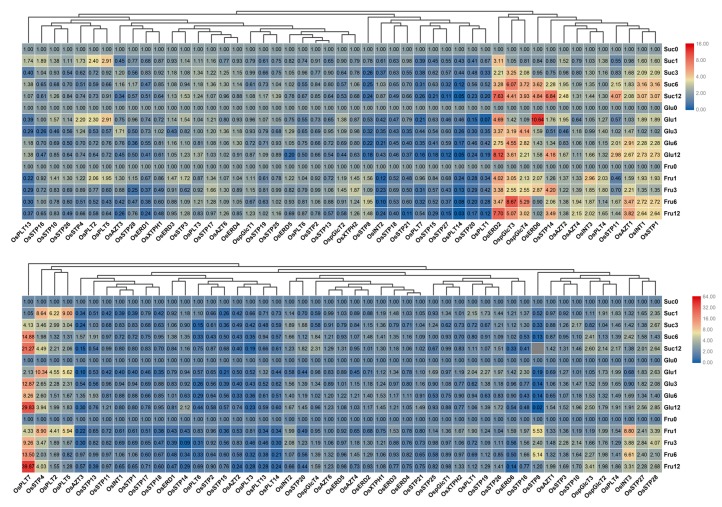
Expression changes of the MST genes in leaf (**A**) and root (**B**) under sucrose, glucose, and fructose treatments by qRT-PCR. 0, 1, 3, 6, 12 represents 0 h, 1 h, 3 h, 6 h, and 12 h after hormone treatment. The fold values were calculated by the 2^−ΔΔCT^ method. The heat map was made based on the fold values. The color scale at the right of each small image represents fold values: red indicates high up-regulation and green represents low up-regulation.

Previous studies reported that MST genes are crucial for various sugar transportations [1,5,8]. We, therefore, detected expression profile of MST genes under 2% sucrose, glucose, and fructose treatments. We found that MST genes showed different expression patterns between leaf and root. In leaf, *OsERD2*, *OspGlcT3*, *OspGlcT4*, *OsERD6*, *OsSTP14*, *OsAZT1*, *OsINT1*, and *OsSTP1* were up-regulated under sucrose, glucose, and fructose treatments. However, *OsAZT2* showed higher up-regulations under sucrose treatment than under glucose and fructose treatments. *OsAZT1* were higher up-regulated under sucrose and glucose treatments than under fructose treatment (Figure 8A). In root, there are lesser up-regulated genes than that in leaf (Figure 8B). *OsPLT7* showed up-regulations under these three sugar treatments. *OsSTP8* and *OsINT3* were only up-regulated under fructose treatment.

### 3.7. Functional Differentiation of MST Duplication Genes under Hormone Treatments and Abiotic Stress Treatments

**Figure 9:**
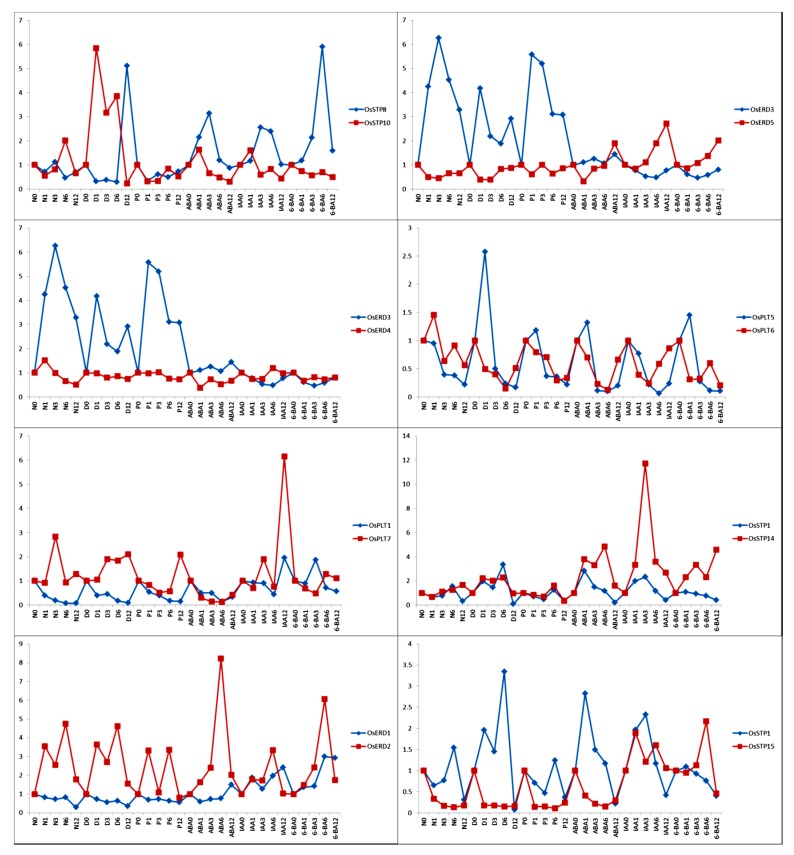
Expression patterns comparison of MST duplicate genes under hormone treatments and abiotic stress treatments. *X*-axis represents the various points from different treatments. *Y*-axis on the left indicates the relative values by qRT-PCR. Red numbers represent significant values.

To investigate the expression patterns of MST duplicate gene pairs and predict functional changes in these genes, we compared the expression patterns of these duplicate gene pairs under hormone treatments and abiotic stress treatments. The duplicate gene pairs are expected to show similar expression patterns [5,8,38]. However, we discovered that only one duplicate gene pair (*OsSTP1* and *OsSTP14*) was ongoing divergent (0.42) and that the remaining duplicate gene pairs were divergent (Figure 9).

## 4. Discussions

In this study, our identification result of MSTs was consistent with previous Johnson’s report [19]. The rice MST gene family, which has 64 genes, is comparable to that in grape (61) [12] and cassava (64) [1], but remarkably greater than that of tomato (49) [11,14] and *Arabidopsis* (53) [9,10,11], and smaller than that of pear (75) [44]. The size of the MST gene family varies between different genomes in a manner independent to their evolution distances, which is the same situation in the MST subfamilies. STP subfamily was the largest subfamily (28) in rice (Figure 1 and Table 1), while it was much smaller in other genomes, such as *Arabidopsis* and cassava. On the other hand, ERD was the largest MST subfamily in *Arabidopsis* while only 6 ERD genes were found in rice (Figure 1 and Table 1) [1,9,10]. These results indicated that MST family had species-specific subfamily expansion in different plants and these expansions were driven by gene duplication events, which might play critical roles in the evolution of MST gene family [1,8,10,18,19,44].

A total of 12 duplication events of rice MSTs were found, including two segmental duplications and ten tandem duplications. However, Johnson et al. (2007) reported 13 duplication events (12 tandem duplications and one segmental duplication) [19], which was a slightly higher frequency than that in our study. This might be due to the use of different softwares and methods. In this present study, we used MCScanX software to detect duplication events, which was used to detected duplication events in the previous studies [38,45]. But our result and Johnson’s result both indicated that tandem duplication plays a more important role than segmental duplication in rice MST family expansions, especially in STP and PLT subfamilies (Figure 3). Interestingly, duplication events were only found in STP, ERD, and PLT subfamilies, which was the same in *Arabidopsis* [19]. Thus, we speculated these expansions of particular subfamilies may be related to the adaption to the terrestrial environment during the process of evolution. This speculation needs further to verification in future work.

The divergence times of these duplication events were estimated to be from 1.80 to 18.08 Mya (Table 2). Previous studies reported that A-genome species of the genus *Oryza* was completed diversification about 5 Mya [46] and the diversification of rice genus and tribe occurred about 14.1 Mya and 23.9 Mya, respectively [47]. In this study, divergence times were estimated to be 18.08 and 10.72 Mya for the two segmental duplications occurred during the evolution of MST family. In addition, the tandem duplication events detected in this study occurred in a wider range of times, 1.80–16.84 Mya. Specifically, four genes were tandemly duplicated at a <5 Mya (after the diversification of *Oryza*) and five genes were tandemly duplicated between the diversification of *Oryza* and rice genus (5 Mya < divergence time < 14.1), one was between the diversification of rice genus and tribe (14.1 < divergence time < 23.9). Based on these data, we summarized that the majority of gene duplications, including tandem duplications and segmental duplications, were produced before diversification of rice genus. During the differentiation process of rice genus, some tandem duplications and segmental duplications also played essential roles in the expansion of MST family. Notably, tandem duplications played a more important role in the recent expansion of A-genome species. Gene structure and conserved motifs of rice MST genes showed the similar characteristics with *Arabidopsis*, cassava, and other species (Figure 4) [1,8,10,44]. These results revealed that MST genes have conserved functions among different species. We found that the intron number of different subfamilies were different. Most MST subfamilies have 3–6 exons except the ERD and XTPH subfamilies and some pGlcT genes. A similar situation was also found in cassava and woodland strawberry [44]. These results indicated that the intron loss/gain might occur before the divergence of monocotyledon and dicotyledon.

Earlier studies proposed that surviving members of gene duplicate pairs usually evolved to neofunctionalization (NF), subfunctionalization (SF), or subneofunctionalization (SNF) [19,48]. As model-1 neofunctionalization, both duplicates retain all original functions (expressions), whereas one duplicate also gains novel functions (expressions) [19]. In this study, different expression patterns were found in all gene pairs (Figure 5). We particularly noticed that *OsSTP14* and *OsSTP1*, which were tandem duplicated genes, showed similar expression patterns in various tissues, except *OsSTP1* was highly expressed in flag leaf and leaf sheath (Figure 5), indicating *OsSTP1* might be model-1 neofunctionalization. In addition, Our results revealed that significant values of most MST duplicate pairs were less than 0.3 and only one duplicate pair (*OsSTP1* and *OsSTP14*) was more than 0.3 (Figure 9). These results suggested that most MST duplicate gene pairs had undergone functional divergence and *OsSTP1* and *OsSTP14* was going divergent. Our expression patterns supported Johnson et al. functional divergence results of rice MST genes that NF, SF, and SNF existed in rice MST genes.

Most MST genes showed tissue-specific expression profiles (Figure 5) except the STP and AZT subfamilies, most of which were ubiquitously expressed. And these results were consistent with previous findings on other species [1,8,10,45]. *OsSTP3* (*OsMST3*) was the first characterized MST gene in rice. Toyofuku et al. (2000) reported that *OsSTP3* was expressed in leaf blades, leaf sheaths and especially high in calli and roots and that OsMST3 was responsible for the accumulation of monosaccharides required for cell wall synthesis [10]. We confirmed its expression in roots and shoots but not in calli, which may be because of the different types of the callus samples. They also reported the characterization of OsSTP1 (OsMST1) and OsSTP2 (OsMST2), which showed no and low D-glucose transport activity, respectively [10]. However, *OsSTP1* was significantly responded in shoots to glucose, fructose and sucrose treatment, which suggested OsSTP1 might play a role in other sugars transportation. *OsSTP4* (*OsMST4*) was a ubiquitous expressed gene with high expression in sink leaf blade, leaf sheath and embryo and OsSTP4 could transport fructose and mannose as well as glucose and galactose [19]. However, *OsSTP4* was relatively low expressed in all tested tissues in our study but highly responded to abiotic stresses and sugar treatments very rapidly, which indicated that OsSTP4 might not be involved in the routine work but in the response to environment change. *OsSTP8* (*OsMST8*) was another low expressed gene in our results but was proved to be very important for the pollen development [49,50]. What’s more, *OsSTP8* was believed to be responded to fructose but its transport activities need further test. Besides, *OsSTP8* was also induced by different hormone treatments in our results and by cold in previous study [50], suggesting *OsSTP8* might also be an important emergency response gene. *OsSTP13* was highly expressed in roots and responded to hormone treatments. *OsSTP27* was highly expressed in shoots and responded to sugar treatments. They showed different expression patterns from their homologs in *Arabidopsis*, *AtSTP3*, which was mainly expressed in leaves and characterized as a H^+^ symporter [51]. *OsSTP10* and *OsSTP14* showed similar expressed patterns and highly response to drought stress, but they showed different response to hormone and sugar treatments. *OsSTP10* did not respond to hormone treatments and was induced in sucrose and fructose treatments in roots. *OsSTP14* responded to ABA, IAA, and 6-BA and was strongly induced in sugar treatments in shoots. *OsSTP16* was mainly expressed in flag leaf sheath and was rapidly responded to glucose and fructose treatments. However, their homologs in *Arabidopsis*, AtSTP5, did not show any monosaccharides transport activities in yeast [10]. Whether OsSTP10, OsSTP14 and OsSTP16 got transport activities towards monosaccharides needs further study. The STP subfamily expanded significantly in rice compared to *Arabidopsis* [18]. The different expression patterns of STP subfamily between species suggested different roles in growth and development, so the OsSTP subfamily needs further work to elucidate their function. The reports on other subfamilies were even less in rice. In previous study, *OsAZT4* (*OsTMT1*) was a ubiquitous expressed gene and *OsAZT1* (*OsTMT2*) showed a similar expression pattern with *OsAZT4* [2]. Our results confirmed these results but *OsAZT1* (*OsTMT2*) showed more redundancy in flag leaf and flag leaf sheath than *OsAZT4* in our study (Figure 5). *OsAZT1* was induced by fructose treatment in shoots, which was consistent with its transport activities towards glucose and fructose [2]. What’s more, *OsAZT1* was strongly induced by sugar and 6-BA treatments, which suggested an important role of OsAZT1 in sugar transport in flag leaf and flag leaf sheath. Besides, *OsAZT2* was strongly expressed in seedlings, indicating its role in early growth and development. Regarding to the pGlcT subfamily, *OspGlcT2* (*OsGMST1*) was a ubiquitous expressed gene with strong response to sugar and salt treatments in our study, which supported its role in tolerance to salt stress [31]. Until now, there are still no reports about PLT, INT, XTPH and ERD subfamilies in rice. The studies in *Arabidopsis* set good examples for the studies in rice. However, even with the closest homologues in the same species, the expression patterns and functions may differ, not to mention that in the different species. The expression results of MST genes in this study provided some important messages for the future functional analysis.

## 5. Conclusions

In our study, a systematic investigation of the OsMST gene family including genome-wide identification, phylogenetic reconstruction, chromosome localization, gene structure analysis, gene duplication events, and Ka/Ks ratios of duplicate gene pairs, was performed. A total of 12 duplicate gene pairs were identified and generated from two segmental duplication and ten tandem duplication events in different evolutionary time points. The qRT-PCR results showed that these duplicate gene pairs have undergone functional divergence. Some MST genes showed upregulation under different abiotic stresses, indicating that these genes may play important roles in abiotic stresses. Among these, *OsERD3* and *OsSTP3* could serve as good candidate genes for improving various stresses tolerance of rice through genetic engineering technologies. In addition, several MST genes were found to respond to different hormone treatments. This work may offer a preliminary insight into the functional variations that have occurred during the evolution of the MST gene family.

## Figures and Tables

**Table 1:** The detailed information on rice monosaccharide transporter (MST) genes.

Name	TIGR Locus Tag	RAP-DB	MST Subfamily
OsAZT1	LOC_Os02g13560.1	Os02g0229400	AZT
OsAZT2	LOC_Os02g58530.1	Os02g0832100	AZT
OsAZT3	LOC_Os03g03680.1	Os03g0128900	AZT
OsAZT4	LOC_Os10g39440.2	Os10g0539900	AZT
OsAZT5	LOC_Os11g28610.1	Os11g0475600	AZT
OsAZT6	LOC_Os11g40540.2	Os11g0620400	AZT
OsERD1	LOC_Os03g24860.1	Os03g0363500	ERD
OsERD2	LOC_Os03g24870.1	Os03g0363600	ERD
OsERD3	LOC_Os05g49260.1	Os05g0567700	ERD
OsERD4	LOC_Os05g49270.1	Os05g0567800	ERD
OsERD5	LOC_Os05g50280.1	Os05g0579000	ERD
OsERD6	LOC_Os11g42430.1	Os11g0643800	ERD
OsINT1	LOC_Os04g41460.1	Os04g0491700	INT
OsINT2	LOC_Os07g05640.1	Os07g0151200	INT
OsINT3	LOC_Os04g43210.1	Os04g0511400	INT
OsSTP1	LOC_Os04g37980.1	Os04g0452700	STP
OsSTP10	LOC_Os02g36414.1	Os02g0573500	STP
OsSTP11	LOC_Os02g36440.1	Os02g0574000	STP
OsSTP12	LOC_Os02g36450.1	Os02g0574100	STP
OsSTP13	LOC_Os03g01170.1	Os03g0101300	STP
OsSTP14	LOC_Os04g37970.1	Os04g0452600	STP
OsSTP15	LOC_Os04g37990.1	Os04g0453200	STP
OsSTP16	LOC_Os04g38010.1	Os04g0453300	STP
OsSTP17	LOC_Os04g38026.1	Os04g0453400	STP
OsSTP18	LOC_Os04g38220.1	Os04g0454200	STP
OsSTP19	LOC_Os06g04900.1	Os06g0141100	STP
OsSTP2	LOC_Os03g39710.1	Os03g0594400	STP
OsSTP20	LOC_Os07g03910.1	Os07g0131200	STP
OsSTP21	LOC_Os07g03960.1	Os07g0131600	STP
OsSTP22	LOC_Os07g10590.1	Os07g0206600	STP
OsSTP23	LOC_Os09g09520.1	Os09g0268300	STP
OsSTP24	LOC_Os09g12590.1	Os09g0297300	STP
OsSTP25	LOC_Os09g15330.1	Os09g0322000	STP
OsSTP26	LOC_Os09g24924.1	Os09g0416200	STP
OsSTP27	LOC_Os10g41190.1	Os10g0561300	STP
OsSTP28	LOC_Os11g38160.1	Os11g0594000	STP
OsSTP3	LOC_Os07g01560.1	Os07g0106200	STP
OsSTP4	LOC_Os03g11900.1	Os03g0218400	STP
OsSTP5	LOC_Os08g08070.1	Os08g0178200	STP
OsSTP6	LOC_Os07g37320.1	Os07g0559700	STP
OsSTP7	LOC_Os01g38680.1	Os01g0567600	STP
OsSTP8	LOC_Os01g38670.1	Os01g0567500	STP
OsSTP9	LOC_Os02g06540.1	Os02g0160400	STP
OspGlcT1	LOC_Os01g04190.2	Os01g0133400	pGlcT
OspGlcT2	LOC_Os02g17500.1	Os02g0274900	pGlcT
OspGlcT3	LOC_Os09g23110.1	Os09g0394500	pGlcT
OspGlcT4	LOC_Os09g27900.1	Os09g0452300	pGlcT
OsPLT1	LOC_Os03g10090.1	Os03g0197100	PLT
OsPLT10	LOC_Os11g41830.1	Os11g0637000	PLT
OsPLT11	LOC_Os11g41840.1	Os11g0637100	PLT
OsPLT12	LOC_Os11g41850.1	Os11g0637200	PLT
OsPLT13	LOC_Os11g41870.1	Os11g0637400	PLT
OsPLT14	LOC_Os12g32760.1	Os12g0512100	PLT
OsPLT15	LOC_Os12g32940.1	Os12g0514000	PLT
OsPLT2	LOC_Os01g73590.1	Os01g0966900	PLT
OsPLT3	LOC_Os10g21590.1	Os10g0360100	PLT
OsPLT4	LOC_Os04g44750.1	Os04g0529800	PLT
OsPLT5	LOC_Os07g39350.1	Os07g0582400	PLT
OsPLT6	LOC_Os07g39360.1	Os07g0582500	PLT
OsPLT7	LOC_Os03g10100.1	Os03g0197200	PLT
OsPLT8	LOC_Os04g58220.1	Os04g0678900	PLT
OsPLT9	LOC_Os04g58230.1	Os04g0679000	PLT
OsXTPH1	LOC_Os03g60820.1	Os03g0823100	XTPH
OsXTPH2	LOC_Os10g42830.1	Os10g0579200	XTPH

TIGR locus tag means gene ID in MSU7.0 (http://rice.plantbiology.msu.edu/), RAP-DB means gene ID in The Rice Annotation Project (RAP, https://rapdb.dna.affrc.go.jp/). MST: monosaccharide transporter.

**Table 2:** Ka, Ks and Ka/Ks values for duplication gene pairs in rice.

Seq_1	Seq_2	Ka	Ks	Ka/Ks	Date (MYA)	Duplication Type
OsSTP10	OsSTP18	0.3290306	0.5779125	0.5693432	18.078602	Segmental duplication
OsERD3	OsERD5	0.1951074	1.1448128	0.1704273	10.720185	Segmental duplication
OsSTP11	OsSTP12	0.0328413	0.1013588	0.3240102	1.8044666	Tandem duplication
OsSTP8	OsSTP7	0.0615979	0.1135565	0.542443	3.3845026	Tandem duplication
OsPLT11	OsPLT12	0.0777016	0.1552719	0.5004226	4.2693165	Tandem duplication
OsERD3	OsERD4	0.0837405	0.5136912	0.1630172	4.6011267	Tandem duplication
OsPLT8	OsPLT9	0.142104	0.3454965	0.4113038	7.807912	Tandem duplication
OsPLT5	OsPLT6	0.1481074	0.3072356	0.4820647	8.1377715	Tandem duplication
OsPLT1	OsPLT7	0.1914638	0.3353385	0.5709568	10.51999	Tandem duplication
OsSTP14	OsSTP1	0.1975753	0.3867837	0.5108159	10.855785	Tandem duplication
OsERD1	OsERD2	0.2101177	0.9200798	0.228369	11.54493	Tandem duplication
OsSTP1	OsSTP15	0.3065527	0.4261786	0.7193058	16.843556	Tandem duplication

Synonymous (Ks) and nonsynonymous (Ka) substitution rates of duplicate gene pairs (Ka/Ks ratios).

## Supplementary Materials

The following are available online at https://www.mdpi.com/2073-4425/10/3/239/s1, Table S1: Primers of MST genes for qRT-PCR in this study. Figure S1. The Maximum Likelihood (ML) tree for MST genes in rice.
