# Supplementary material for: A Novel Insight into Functional Divergence of the MST Gene Family in Rice Based on Comprehensive Expression Patterns
Source: Genes (Basel). 2019 Mar 20;10(3):239. doi: 10.3390/genes10030239 (PMC6470851; doi:10.3390/genes10030239)
Supplement: Supplementary file 1 [file genes-10-00239-s001.pdf]

Table S1. Primers of MST genes for qRT-PCR in this study.

Primers	5′–3′	Tm	Position	Length	Annealing Temperature
MST1-F	TCTTCATCGCCGTCTTC	58.9	1576	87	58
MST1-R	GCTTCCAGTACCAGTGT	59.8	1662		
MST2-F	TGACCGTCGTGTGGAGAA	64.8	1599	121	63
MST2-R	ATCAGACAGCAGTGGCATTG	64.9	1719		
MST3-F	TGGTGCTCGTCTGGAA	61.4	1590	169	63
MST3-R	GGATGAACACAAGAAGAAGAATATAG	61.7	1758		
MST4-F	CCATCTTCGCCTTCTTCTC	61.8	1346	104	58
MST4-R	CCTCTCGGTCATCTCCTC	62	1449		
MST8-F	GTGGTGGTGGTGTTCAAC	62.7	1282	144	62
MST8-R	CGGCAGGAAGAAGAAGAC	61.6	1425		
MST10-F	CGCTGGTGTGCCTGTA	63.1	1196	163	62
MST10-R	CGGAGGAACGACTGTGT	62.7	1358		
MST11-F	GCAGTCGCAGTCATTCTT	61.9	1446	85	62
MST11-R	CGTCATCATCACCAACCAT	61.7	1530		
MST13-F	CCTGCCTCTGCGTCAT	62.9	1379	163	63
MST13-R	TGGTGGTGGTGGTGTAG	62.3	1541		
MST14-F	CGGTGGAGGTGAGGTC	62.4	1283	127	65
MST14-R	GCGTAGTAGGCGAATGTG	62.1	1409		
MST15-F	CGACGATGGCGAAGAG	61.1	1127	165	61
MST15-R	TGAGCGAGACGGACAG	62	1291		
MST16-F	CGGTTGAGGTCAGGTC	60.1	1103	128	62
MST16-R	GGCGTAGAAGAGGAACAC	61.2	1230		
MST17-F	ACGGCACCTTCCTCTT	61.5	1367	156	62
MST17-R	CGAACTTGTGGTCCTTGA	61	1522		
MST18-F	TCGTCATCGCCTTCTTC	60.4	905	105	62
MST18-R	TGGAGCCGAGGTTGA	60.5	1009		
MST19-F	CCAGCGAGGTGTTCC	60.3	1256	145	62
MST19-R	GCGGCGAAGAAGAAGA	60.5	1400		
MST20-F	ACCGCCTTCGTCTACC	62	1132	136	61
MST20-R	CTCCGCCTTCCTCCTC	62.1	1267		
MST21-F	CGTGGCGGTGAACTTC	62	1302	139	65
MST21-R	CGGCAAGAGGAGGTAGA	61.4	1440		
MST25-F	TGTCCATCTTCGTCATCCT	62	1403	92	62
MST25-R	CTTCCAATACCAGTGCTTGT	62	1494		
MST26-F	CTCTTCTTCACCTTCGTCATC	62.2	1297	182	63
MST26-R	AACCAGTGCTTCCTCCATA	62.2	1478		
MST27-F	GCAGAGTTCAAGGACTATGG	61.9	1129	152	63
MST27-R	TCTCAAGTGGACATATCTCAGT	62.3	1270		
MST28-F	GGATGAAGGCGTGGAT	59.8	1379	136	61
MST28-R	CAGAACCAGTGCTCCTC	60.6	1514		
OspGlcT1-F	ACAAGCAAGGAAGGAAGAG	61.2	1247	128	62
OspGlcT1-R	GCCAGCAACAGCAAGA	61.5	1374		
OspGlcT2-F	TTCGTTAGGAGCAGGTC	59.5	1164	143	60
OspGlcT2-R	CCAGAAGCCGCAAGA	59.8	1306		
OspGlcT3-F	AGTTACAGGAATCATCATACCA	60.6	1365	187	62
OspGlcT3-R	AAGTGCTGACAACAAGGA	60.5	1551		
OspGlcT4-F	CAGTTACAGGAATCATCATACCA	61.9	455	188	61
OspGlcT4-R	AAGTGCTGACAAGAAGGATAC	61.8	642		
OsAZT1-F	CAATCAGCGTCATCATCTACTT	62.4	1904	142	62
OsAZT1-R	ATATCACCAATCCAGAATGTCAG	62.2	2045		
OsAZT2-F	GGAGCGTGGTGGTGTA	62.3	1607	121	63
OsAZT2-R	GCGAGCGAGCAGATG	61.4	1727		

OsAZT3-F	TGCTTCCTTCTATTGGTATTG	59.5	1637	135	60
OsAZT3-R	TTGCTTCTAAGTTCATAACATTG	59.3	1771		
OsAZT4-F	GCGACAAGCGTGGAAG	62.4	1129	138	63
OsAZT4-R	CCAAGCCAACTGCCATC	62.5	1266		
OsAZT6-F	TTGCTCACTTGCCTTCTG	61.7	1752	155	63
OsAZT6-R	CCTTCGTCTCTGGAACTCT	62.3	1906		
OsERD1-F	CTTGGTGACGCTGGTTAG	61.9	1410	107	63
OsERD1-R	TCGCCGCAGAGAACA	61.8	1516		
OsERD2-F	AGTTAGTTGGTTAGGTTCCTTC	61.6	1341	176	58
OsERD2-R	TGAGCGAGTCCTGAATCT	61.5	1516		
OsERD3-F	GGCAATAACAATGACAGCAA	61.1	1335	102	58
OsERD3-R	AATATGACGAACACGAGAGT	60.5	1436		
OsERD4-F	GACGCTCGCCAACTG	61.5	1320	183	63
OsERD4-R	CGGAAGGACCATTGTATCTC	61.6	1502		
OsERD5-F	CATAATGTCTGAGATTCTTCCT	59.2	1266	153	61
OsERD5-R	ACAGCAGCATAGATAGCA	59.6	1418		
OsERD6-F	GATGGACAGAAGTGGTAGAAG	61.6	945	138	58
OsERD6-R	GTGAGTGCGATGATTGGTA	61.4	1082		
OsXTPH1-F	CCACTACTTATAGGAGGCATTG	61.9	1237	101	63
OsXTPH1-R	GCACCAACAGCGACAA	61.8	1337		
OsXTPH2-F	TTATGCTGCGACAATCCT	60.5	978	162	61
OsXTPH2-R	CCACTAACACCTCCAATCA	60.6	1139		
OsPLT1-F	CTTCCTCTCGCTCTCCAA	62.2	1329	168	63
OsPLT1-R	TCATCGTCGTCGTCTGT	61.7	1496		
OsPLT2-F	CCAGCGGCGTCATCT	62.8	1130	120	58
OsPLT2-R	GGAGGTAGGTGAAGAAGAAGA	62.4	1249		
OsPLT3-F	CGTTCGTGTCGCTCTA	59.8	1283	99	58
OsPLT3-R	GGCACAGGAGGTAGAAG	60	1381		
OsPLT4-F	GGAGCAGATTGAGATGATGTT	62	1458	113	63
OsPLT4-R	AGCCTAGAGACACAGACTTC	62.4	1570		
OsPLT5-F	CAGCGGCGTCATCTC	60.9	1494	119	61
OsPLT5-R	GGAGGTAGGTGTAGAAGAAC	60.5	1612		
OsPLT6-F	TCTACGCCGCCATCT	60.8	1340	166	58
OsPLT6-R	GGAGAAGTTGCCAAGAGT	60.7	1505		
OsPLT7-F	GGCACGGCGATGAAC	62	1291	144	63
OsPLT7-R	CGGCAGGAAGAAGAACAT	61.2	1434		
OsPLT13-F	CGGCAGCGAGATCAT	59.7	1188	150	58
OsPLT13-R	CCAGCGTACACGAAGA	59.7	1337		
OsPLT14-F	GCGTGTCGTCGTCTT	60.3	1151	140	62
OsPLT14-R	CAGCGGCAGGATCTC	60.1	1290		
OsINT1-F	CTTCATCTTCGTGGCTCTC	61.8	1392	110	62
OsINT1-R	GGCTCTGGCGATTGC	61.7	1501		
OsINT2-F	GCGACGGCGAACTG	61.4	1513	134	58
OsINT2-R	CAGACGAGCACGAACG	61.7	1646		
OsINT3-F	CCACCTTCTTCCTCTTCTG	60.9	1556	128	62
OsINT3-R	CGGCTTGTAGTCCTTCTC	60.9	1683		

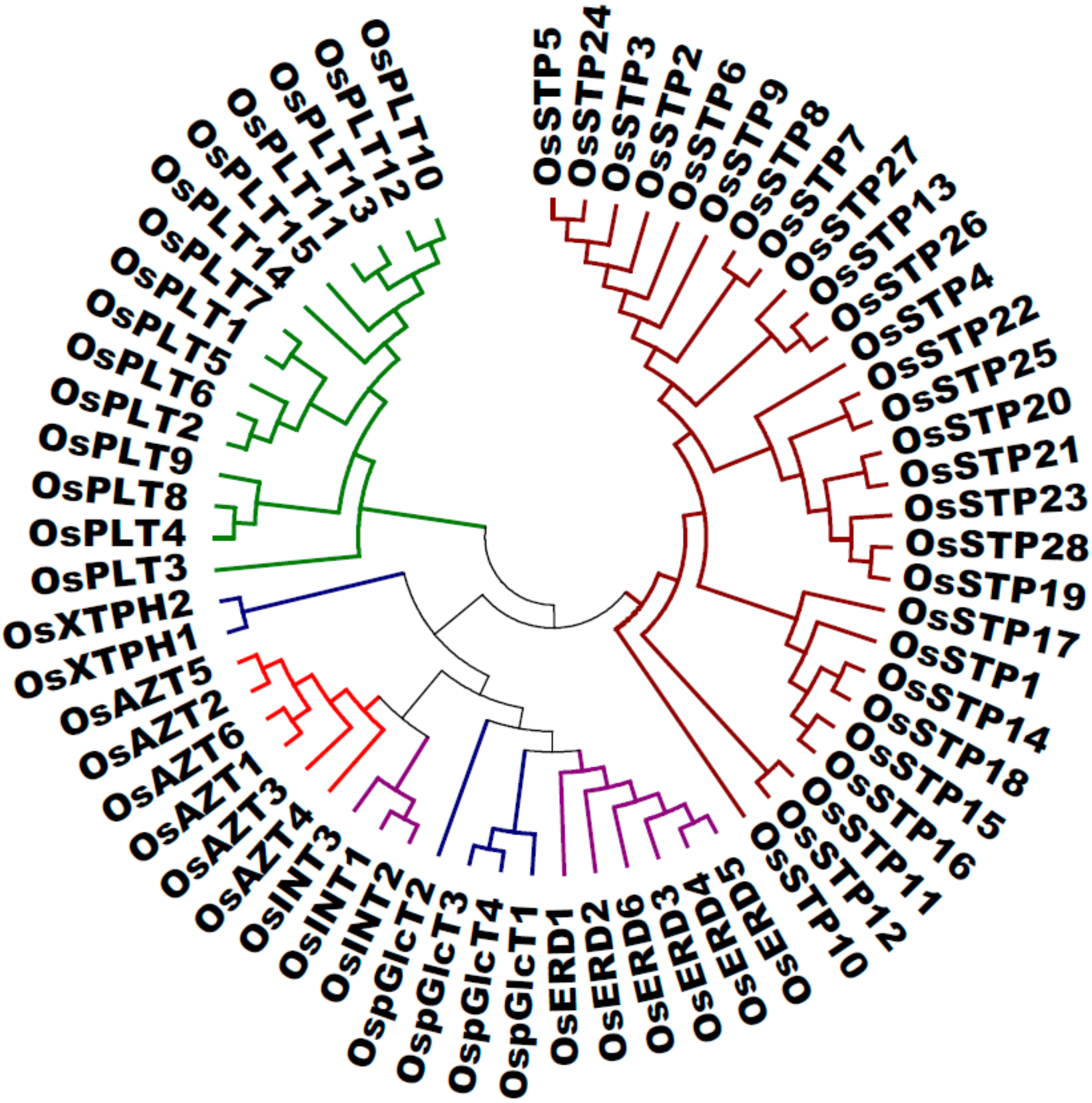

Figure S1. The Maximum Likelihood (ML) tree for MST genes in rice.
